# Urban traffic-derived nanoparticulate matter reduces neurite outgrowth via TNFα in vitro

**DOI:** 10.1186/s12974-016-0480-3

**Published:** 2016-01-26

**Authors:** Hank Cheng, David A. Davis, Sina Hasheminassab, Constantinos Sioutas, Todd E. Morgan, Caleb E. Finch

**Affiliations:** Davis School of Gerontology, University of Southern California, Los Angeles, CA 90089 USA; Viterbi School of Engineering, University of Southern California, Los Angeles, CA 90089 USA; USC Dornsife College, University of Southern California, Los Angeles, CA 90089 USA

**Keywords:** Air pollution, Nanoparticulate matter, Olfactory neuroepithelium, TNFα, Neurite outgrowth, Cell culture, Microglia

## Abstract

**Background:**

The basis for air pollution-associated neurodegenerative changes in humans is being studied in rodent models. We and others find that the ultrafine particulate matter (PM) derived from vehicular exhaust can induce synaptic dysfunction and inflammatory responses in vivo and in vitro. In particular, a nano-sized subfraction of particulate matter (nPM, PM0.2) from a local urban traffic corridor can induce glial TNFα production in mixed glia (astrocytes and microglia) derived from neonatal rat cerebral cortex.

**Methods:**

Here, we examine the role of TNFα in neurite dysfunctions induced by nPM in aqueous suspensions at 12 μg/ml. First, we show that the proximal brain gateway to nPM, the olfactory neuroepithelium (OE), rapidly responds to nPM ex vivo, with induction of TNFα, activation of macrophages, and dendritic shrinkage. Cell interactions were further analyzed with mixed glia and neurons from neonatal rat cerebral cortex.

**Results:**

Microglia contributed more than astrocytes to TNFα induction by nPM. We then showed that the threefold higher TNFα in conditioned media (nPM-CM) from mixed glia was responsible for the inhibition of neurite outgrowth by small interfering RNA (siRNA) TNFα knockdown and by TNFα immunoneutralization. Despite lack of TNFR1 induction by nPM in the OE, experimental blocking of TNFR1 by TNFα receptor blockers restored total neurite length.

**Conclusions:**

These findings implicate microglia-derived TNFα as a mediator of nPM in air pollution-associated neurodegenerative changes which alter synaptic functions and neuronal growth.

## Background

Air pollution epidemiology has traditionally focused on cardiovascular and respiratory outcomes. These adverse associations have been extended to show the acceleration of cognitive decline of elderly community-based populations [1–5] and neurodevelopmental impairments of children [6, 7]. The causes of cognitive impairment are being analyzed in rodent and cell models, which implicate neuroinflammatory responses to urban air pollutants [8–11]. Specifically, we and others observed that the ultrafine size class of air pollution PM0.2 (<0.2 μm diameter) activated microglia and induced TNFα and IL-1, among other inflammatory responses [10, 12–14]. This evidence supports findings of increased microglial activation and white matter hyperintensities in small postmortem samples of children from a highly polluted Mexican city [7, 15] and in the association of white matter loss in older human adults in an MRI analysis of the WHIMS cohort of US women [16].

We focus on traffic-derived ultrafine PM, which consistently shows higher toxicity than larger PM in vivo and in vitro [17, 18], in neonatal rodents. Artificial ultrafine PM is rapidly transported after inhalation into the brain via the olfactory pathway [19, 20]. Within the ultrafine PM, we examined a subfraction eluted from filters into aqueous suspension for its neurotoxicity and pro-inflammatory activity [10, 11]. This subfraction is designated as nano-sized PM (nPM) to distinguish it from the total ultrafine PM and is depleted in black carbon and water-insoluble organics (Table 1) [10]. The nPM fraction is highly active in vitro and in vivo after re-aerosolization, with free radical EPR signals that persisted >30 days after initial collection. Notably, ozone and other gaseous pollutants with epidemiological cognitive associations [2, 21] are absent from filter-collected nPM. In rodent cell models, nPM has both direct and indirect effects on neuronal viability and neurite outgrowth [10]. Because TNFα is induced by chronic inhalation of ultrafine PM [8, 22] and because TNFα can alter neurite outgrowth [23–25], we further evaluated the role of TNFα in rapid brain responses to nPM. We first investigated the olfactory epithelium (OE), since little is known about the initial cellular responses of the olfactory gateway to urban traffic-derived ultrafine (or, equivalently) nPM. Based on the precedent of ex vivo OE incubation from OE biopsies [26, 27], we developed an ex vivo model for incubation of the intact OE within neonatal mouse nasal cavities with nPM suspensions. In addition, using glia and neurons derived from neonatal cerebral cortex, we analyzed mechanisms by which nPM-induced TNFα inhibits neurite outgrowth.

**Table 1:** Composition of nPM

	Ambient nPM (%)	Eluted nPM (%)	% ambient in eluted nPM
Black carbon	13	1	7
Organic carbon, water soluble	32	34	100
Organic carbon, water insoluble			
Hopanes-steranes	0.012	0.001	8.5
Organic acids	0.097	0.009	9
Polyaromatic hydrocarbons	0.02	Not detected	0
Metals (Cu, Fe, Ni, V)			>90

Content of black carbon, water-soluble and water-insoluble organic carbons, and metals in ambient nPM compared with eluted filter-trapped nPM. Percent recovery of ambient nPM in eluted samples was calculated to show efficiency of transfer. Data derived from [10]

## Methods

### nPM collection and transfer into aqueous suspension

Nano-sized particulate matter (nPM; <0.2 μm in diameter) was collected on Teflon filters by a High-Volume Ultrafine Particle (HVUP) Sampler [28] at 400 l/min flow in urban Los Angeles, downwind from the local I-110 Freeway [10]. These samples are a mix of fresh ambient PM, mostly from vehicular traffic emissions and secondary aerosols [29, 30]. The nPM samples were collected continuously during July–Sept. 2010 and Nov. 2011–Feb. 2012; these pooled samples approximate the annual average composition of nPM near the I-110 corridor [31]. The filter-trapped dried nPM were eluted by sonication into deionized water. The nPM comprise 20 % by mass of ambient PM2.5. Water-soluble metals and organic compounds were efficiently transferred (Table 1). Relative to the total filter-trapped ultrafines (PM0.2), the nPM subfraction eluted into aqueous phases is depleted in black carbon and water-insoluble organic compounds. nPM suspensions (350 μg/ml) were stored at −20 °C. For controls of nPM extracts, fresh sterile filters were sham-extracted.

### Animals

C57BL/6J mice were purchased from The Jackson Laboratory (Sacramento, CA, USA) for breeding and pregnant Sprague Dawley rats from Harlan Labs (Livermore, CA, USA). Animals were maintained following NIH guidelines, approved by the USC Institutional Animal Care and Use Committee (IACUC). Animals were euthanized by cervical dislocation after anesthesia by isoflurane or CO_2_.

### Nasal cavity ex vivo incubation

P3 mice (both sexes) were anesthetized and decapitated; the nasal bone was removed to reveal the nasal cavity. The entire nasal cavity including the snout intact was removed in the gross. Nasal cavities were incubated with 12 μg/ml nPM in artificial cerebral spinal fluid (CSF) for 2 h/37 °C. After incubation, the OE was peeled from the nasal cavity for quantitative polymerase chain reaction (qPCR) or immunohistochemistry. Mice were chosen for these experiments because their smaller size facilitates slide preparation and obviates decalcification.

### Cell culture

Mixed glia were originated from the cerebral cortex of postnatal day 3 (P3) rats (both sexes). Primary glia were grown in Dulbecco’s modified Eagle’s medium/Ham’s F12 50/50 Mix (DMEM F12 50/50) supplemented with 10 % fetal bovine serum (FBS) and 1 % l-glutamine in a humidified incubator (37 °C/5 % CO_2_) [32]. After culture for 2.5 weeks, their composition was 3:1 astrocytes:microglia. Microglia were isolated by shaking for 4 h/37 °C. Embryonic day 18 (E18) rat cortical neurons were originated at 15,000 neurons/cm^2^ on poly-d-lysine-coated coverslips in DMEM supplemented with B27 (Invitrogen, Grand Island, NY).

For in vitro exposure, mixed glia were trypsinized and replated in six-well plates at 1 × 10^6^ cells/well and grown overnight. Secondary cultures of mixed glia were treated with nPM aqueous suspensions (12 μg/ml) diluted in neuronal media for 24 h before assay. This dose consistently induced glial TNFα and IL-1α messenger RNA (mRNA) [10]. The resulting conditioned media (CM) was collected and centrifuged (10,000*g*/10 min) to remove residual cells. For small interfering RNA (siRNA) experiments, mixed glia were treated with siRNA (Silencer Negative Control No. 1 siRNA, AM4611; Ambion, Austin, TX) or TNFα siRNA (AM16708, Ambion). Scrambled and TNFα siRNAs were mixed with a siPORT NeoFX transfection agent (Ambion) to 50 nM. Mixed glia were grown for 24 h post transfection and then treated with nPM or vehicle before plating onto E18 neurons. Immunoneutralization of TNFα used 20 μg/ml antibody (MAB510; R&D Systems, Minneapolis, MN); TNF receptor activity was inhibited by TNFR1/2 blocking peptide (E-20, L-20; SCBT, Dallas, TX) at 5 μg/ml before CM application. Rats were used for in vitro experiments, following our prior studies [10] and the better yields of microglia than from mice.

### Quantitative polymerase chain reaction

Total cellular RNA was extracted using TRI reagent (Sigma, St. Louis, MO). cDNA was prepared from 1 μg of RNA by Superscript III RT kit (Invitrogen, Carlsbad, CA) and analyzed by qPCR with appropriate primers for both mouse and rat for Ct (threshold cycle) values. Genes examined by qPCR include TNFα (forward: 5′ CGTCAGCCGATTTGCTATCT 3′; reverse: 5′ CGGACTCCGCAAAGTCTAAG 3′) (CT range 26–30), Iba1 (forward: 5′ CCTGATTGGAGGTGGATGTCAC 3′; reverse: 5′ GGCTCACGACTGTTTCTTTTTTCC 3′) (CT range 25–26), IL-1α (forward: 5′ TCGGGAGGAGACGACTCTAA 3′; reverse: 5′ GTGCACCCGACTTTGTTCTT 3′) (CT range 29–31), GFAP (forward: 5′ CCAAGCCAAACACGAAGCTAA 3′; reverse: 5′ AGGAATGGTGATGCGGTTTTC 3′) (CT range 30–31), iNOS (forward: 5′ CATTGGAAGTGAAGCGTTTCG 3′; reverse: 5′ CAGCTGGGCTGTACAAACCTT 3′) (CT range 27–29), TNFR1 (forward: 5′ GGGCACCTTTACGGCTTCC 3′; reverse: 5′ GGTTCTCCTTACAGCCACACA 3′) (CT range 22–23), TNFR2 (forward: 5′ CAGGTTGTCTTGACACCCTAC 3′ reverse: 5′ GCACAGCACATCTGAGCCT 3′) (CT range 25–26), βIII-tubulin (forward: 5′ CGCACGACATCTAGGACTGA 3′; reverse: 5′ TGAGGCCTCCTCTCACAAGT 3′) (CT range 19–20), and rGAPDH (forward: 5′ AGACAGCCGCATCTTCTTGT 3′; reverse: 5′ CTTGCCGTGGGTAGAGTCAT 3′) (CT range 16–17). Data were normalized to GAPDH and quantified as ΔΔCt.

### ELISA

CM from nPM-treated glia was sampled after 24 h of exposure and analyzed for TNFα by solid phase sandwich ELISA (BD Biosciences, San Jose, CA).

### Immunohistochemistry

The OE and olfactory bulb of P3 neonatal mice were fixed with 4 % paraformaldehyde in phosphate buffered saline (PBS) pH 7.4. Specimens were immersed in 10 % sucrose/PBS pH 7.4, then 30 % sucrose/PBS pH 7.4 at 4 °C, then embedded in optimal cutting temperature compound (OCT; Fisher Scientific, Waltham, MA) before transverse cryostat sectioning (18 μm). Antigen retrieval was performed by submerging slides in 10 mM sodium citrate buffer and microwaving for 3 min. Tissue was permeabilized with 1 % NP-40/PBS and blocked with 5 % BSA, then probed with antibodies specific for the Olfactory Marker Protein of olfactory sensory neurons (OMP 1:100; SCBT, Dallas, TX), βIII-tubulin (1:400; Sigma Chemical Co., St. Louis, MO), astrocytes (GFAP 1:400; Sigma), and microglia (Iba1 1:200, Wako). Immunofluorescence was visualized with Alexa Fluor 488 or 594 antibodies (1:400; Molecular Probes).

### Microscopy

Fluorescent images were analyzed with a Nikon Eclipse TE300 microscope (Nikon, Melville, NY). One hundred neurons were selected from a distribution of nine images per coverslip for analysis.

### Neurite outgrowth assays

After exposure to glial conditioned media, E18 neurons were fixed in 4 % paraformaldehyde and immunostained with anti-βIII-tubulin (1:400). Neurites were visualized by F-actin with Rhodamine phalloidin (1:50; Molecular Probes, Carlsbad, CA). Images were analyzed for neurite length, density, and number by NeuronJ of ImageJ software; soma size was determined by the Neurphology plugin of ImageJ. Only neurons with neurites fully visible were analyzed. Neurite density was assayed as total βIII-tubulin fluorescence after skeletonizing. Axons were identified as the longest neurite [33].

### Image analysis

The olfactory sensory neuron (OSN) dendritic layer of the OE was assessed by NeuronJ plugin of ImageJ in 20 evenly spaced regions in the nasal septum and ethmoturbinates. The dendritic layer thickness was defined as the distance between the OSN cell body and the outer edge of the sensory dendrites in the nasal cavity.

### Statistical analysis

GraphPad Prism Version 5 (Graph Pad, La Jolla, CA) was used. Single and multiple comparisons used Student’s *t* test (unpaired) and ANOVA/Tukey’s multiple comparison post-test, respectively. Level of significance alpha = 0.05.

## Results

### nPM rapidly induced TNFα in olfactory neuroepithelium ex vivo

**Fig. 1:**
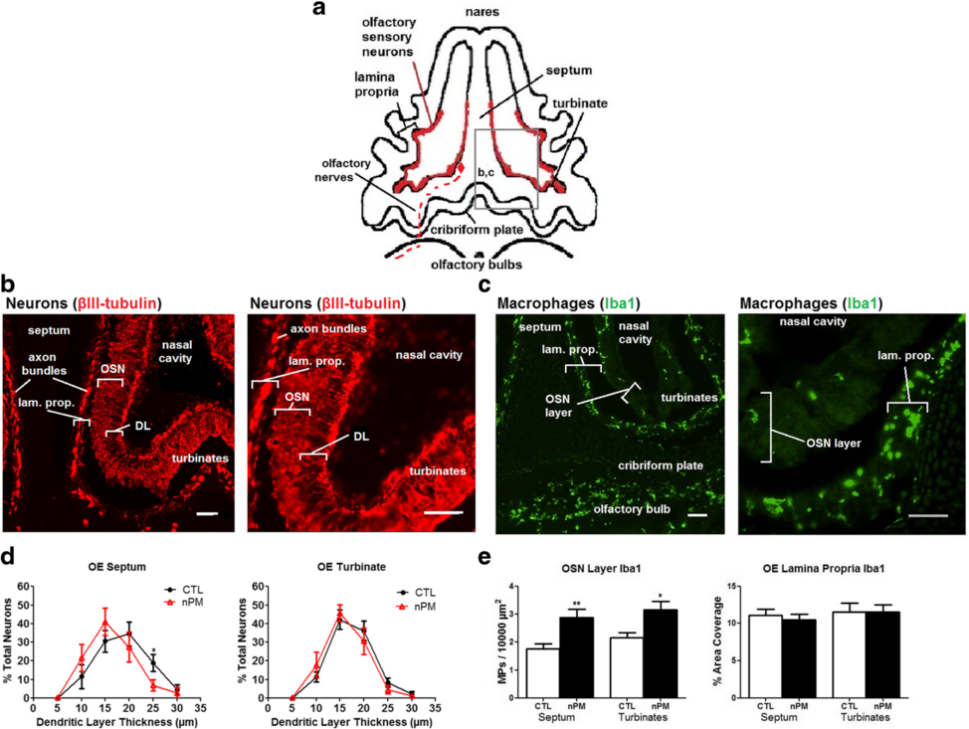
Anatomical schema and cell types in neonatal mouse olfactory neuroepithelium. **a** Transverse section of neonatal mouse nares to show the nasal cavity, cribriform plate, and olfactory bulb (OB). Olfactory sensory neurons (OSN, *red*) are within the lamina propria mucosal layer of the medial nasal septum and ethmoturbinates. OSN axon bundles project to the OB through the cribriform plate as olfactory nerves (*red dashed line*; *red diamond*, perikarya). **b, c** *Box outlines* locate IHC images. **b** OE turbinates and medial septum contain βIII-tubulin positive OSNs. The larger magnification of the turbinate region shows the dendritic layer (DL) and the OSN axon bundles in the lamina propria (*lam. prop.*). *Scale bar =* 50 μm. **c** Iba1-immunopositive macrophages in OE form a dense layer in the lamina propria. **d** Thickness of the dendritic layer in the OE turbinate and septum in response to incubation with nPM. The OE septal dendrites showed a 10 % shrinkage in response to nPM, with consistent shift across all size classes in the frequency distribution (*left panel*; *p < 0.05; *t* test; *n* = 8 nares per group; 30 DL thickness measurements per condition); the means did not differ significantly (CTL 18.0 ± 0.95 μm, nPM 16.2 ± 1.01 μm). The turbinate dendritic layer did not respond to nPM. **e** Macrophage numbers (Iba1-immunopositive) in the OSN were increased 50 % by nPM, with no change of total Iba1 staining within the lamina propria (*p < 0.05, **p < 0.01; *t* test). nPM treatment did not detectibly alter Iba1 staining or MP cell morphology

First, we characterized the OE cell populations which are incompletely described for neonatal mice. Figure 1a represents the main anatomic features of the nasal cavity. The OE contains well differentiated OSN (βIII-tubulin-immunopositive) with perikarya on the inner face of the OE (Fig. 1b); the OSN dendrites extend into the mucosa lining the turbinate space as a layer distinct from their perikarya; axon bundles of the OSN project through the cribriform plate to the olfactory bulb (OB), identified by immunostaining for the OSN-specific Olfactory Marker Protein (not shown). Macrophages (Iba1-immunopositive) defined a dense layer in the lamina propria, sharply demarcated from their lower density in the adjacent OSN layer/lamina propria (Fig 1c). GFAP immunostaining for astrocytes was not above background in the OE; in contrast, the OB has numerous GFAP-positive astrocytes (not shown).

**Fig. 2:**
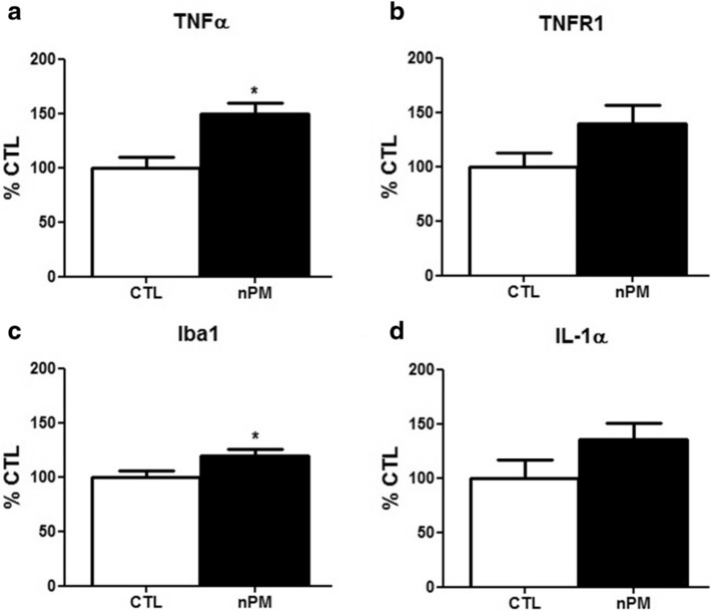
nPM rapidly induced cytokine mRNA in ex vivo olfactory neuroepithelium. **a**, **c** Incubation with nPM (12 μg/ml, 2 h) increased TNFα mRNA by 50 % and Iba1 by 20 % (*p < 0.05; *t* test; *n* = 7). **b**, **d** IL-1α and TNFR1 (*p* = 0.079, 0.058, respectively). GFAP mRNA was below reliable PCR values (OE Ct >30 vs cerebral cortex Ct <24, positive control)

As an ex vivo model for the initial contact of brain with nPM, the nasal cavities of neonatal mice were incubated with nPM suspensions. Ex vivo nasal cavities were incubated with 12 μg/ml nPM for 2 h, conditions that induced TNFα in mixed glial cultures [10]. The OSN responded with shrinkage of the dendritic layer in the OE septal zone by 10 % across the dendritic length frequency distribution; turbinate zone dendrites had smaller responses (Fig. 1d). Macrophages also responded, with a 50 % increase in Iba1-immunopositive cells in the OSN layer (Fig. 1e). By qPCR, we found increased levels of inflammation-related mRNAs in the OE including 45 % increase of TNFα mRNA and 20 % increase of Iba1 mRNA (Fig. 2a, c), with trends for increased IL-1α and TNFR1 (Fig. 2b, d); definitive non-changers included iNOS, TNFR2, and βIII-tubulin mRNA (not shown). GFAP mRNA was below reliable CT values (see Fig. 2 legend), consistent with background GFAP immunostaining in the OE noted above. Because TNFα inhibits neurite outgrowth in vitro and in vivo [24, 34], we hypothesized that TNFα secreted by OE macrophages is an initial event in neurite shrinkage.

### nPM-induced TNFα in both astrocytes and microglia

**Fig. 3:**
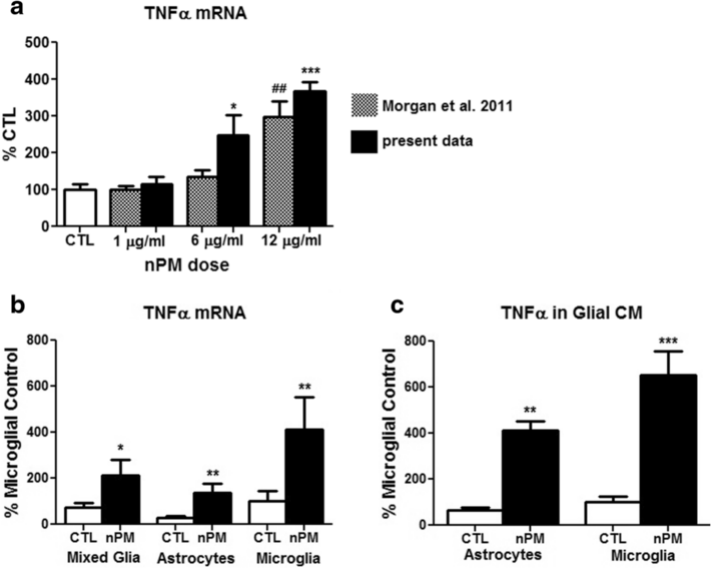
Mixed glia exposed to nPM: increased TNFα mRNA and protein. **a** TNFα mRNA was significantly induced by 6 and 12 μg/ml nPM. (*p < 0.05, ##*p* < 0.01, ***p < 0.001; ANOVA with Tukey’s post-test; *n* = 6). Prior data (*cross-hatched*) was shown to document equivalent activity of different samples of nPM collected at the same site on different years (2009 vs 2010–2011). **b** TNFα mRNA in mixed glia, enriched astrocytes, and enriched microglia treated with nPM were increased by >3-fold vs CTL: microglia > mixed glia > astrocytes (*p < 0.05, **p < 0.01; *t* test; *n* = 9). Treated microglia had the lowest base Ct values. **c** TNFα protein in CM was increased by fivefold vs CTL (**p < 0.01; ***p < 0.001; *t* test; *n* = 6)

To facilitate analysis of relationships between the glial secretion of TNFα and neurite length, we used mixed glial cultures from the cerebral cortex of neonatal rat, in which TNFα mRNA was readily induced by nPM (Fig. 3a). The dose response reported for a prior sample of nPM collected in January 2009 [10] was closely matching. Extending these findings, exposure to nPM at 12 μg/ml for 24 h induced TNFα mRNA >3-fold in cultures of separated microglia or astrocytes (Fig. 3b). Microglial responses were larger than astrocytes by the TNFα/GAPDH ratio. The CM from mixed glia that were exposed to nPM (nPM-CM) showed corresponding increases in TNFα protein, again with greater increases from microglia (Fig. 3c). Cell levels of TNFα mRNA and of CM TNFα protein were positively correlated (*r*^2^ = 0.28, not shown). Because glial gene expression can depend on contact between microglia and astrocytes, e.g., apolipoprotein E and apolipoprotein J [35, 36], it is notable that the TNFα/GAPDH in separated astrocytes (0.0048) and microglia (0.0145) approximated that of mixed glia (0.0755) after adjusting for their relative proportions in mixed glia. Despite their minority as ~25 % of the cells in mixed glia, microglia contributed 60 % of the TNFα protein in CM.

### Conditioned media from nPM-treated astrocytes and microglia reduce neurite outgrowth

**Fig. 4:**
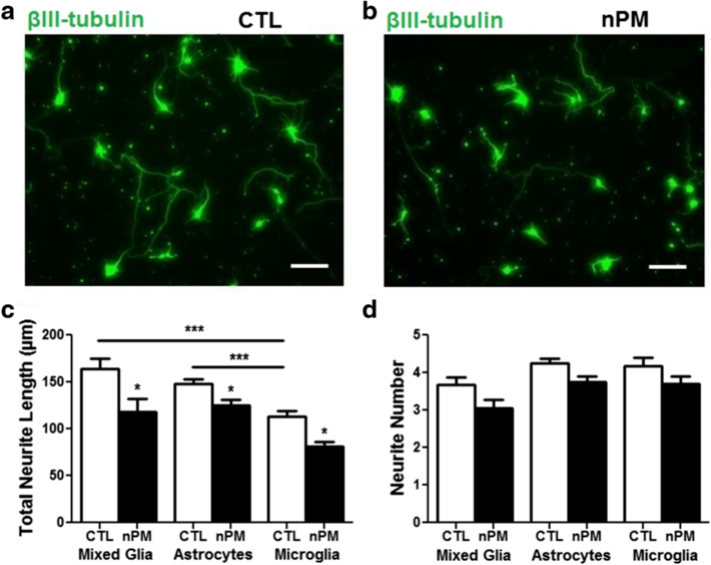
CM from glia treated with nPM decreased neurite outgrowth. **a** Neurons treated with mixed glial CM; βIII-tubulin IHC; *scale bar* = 40 μm. **b** Neurons treated with CM from nPM-treated mixed glia; βIII-tubulin IHC; *scale bar* = 40 μm. **c** Addition of CM from nPM-treated mixed glia, astrocytes, or microglia decreased neurite length by 20, 15, and 30 %, respectively, vs controls. Microglial CM controls had lower baseline neurite outgrowth compared to mixed glia CM and astrocyte CM. (*p < 0.05, ***p < 0.001; ANOVA with Tukey’s post-test; *n* = 100 neurons). **d** Neurite number showed trend for decrease by addition of CM from nPM-treated mixed glia, astrocyte, or microglia (n.s., ANOVA)

nPM-CM from mixed glia or enriched astrocytes and microglia was analyzed for neurotrophic activity by neurite outgrowth of E18 rat cerebral cortex neurons and supported less neurite outgrowth, assessed by length: mixed glia, −20 %; astrocytes, −15 %; microglia, −30 % (total neurite length per neuron) (Fig. 4a–c) with a trend for fewer neurites (Fig. 4d). Neurons grown in microglial CM had lower baseline neurite outgrowth (Fig. 4c).

### Inhibiting or reducing TNFα in the CM rescued neurite outgrowth

**Fig. 5:**
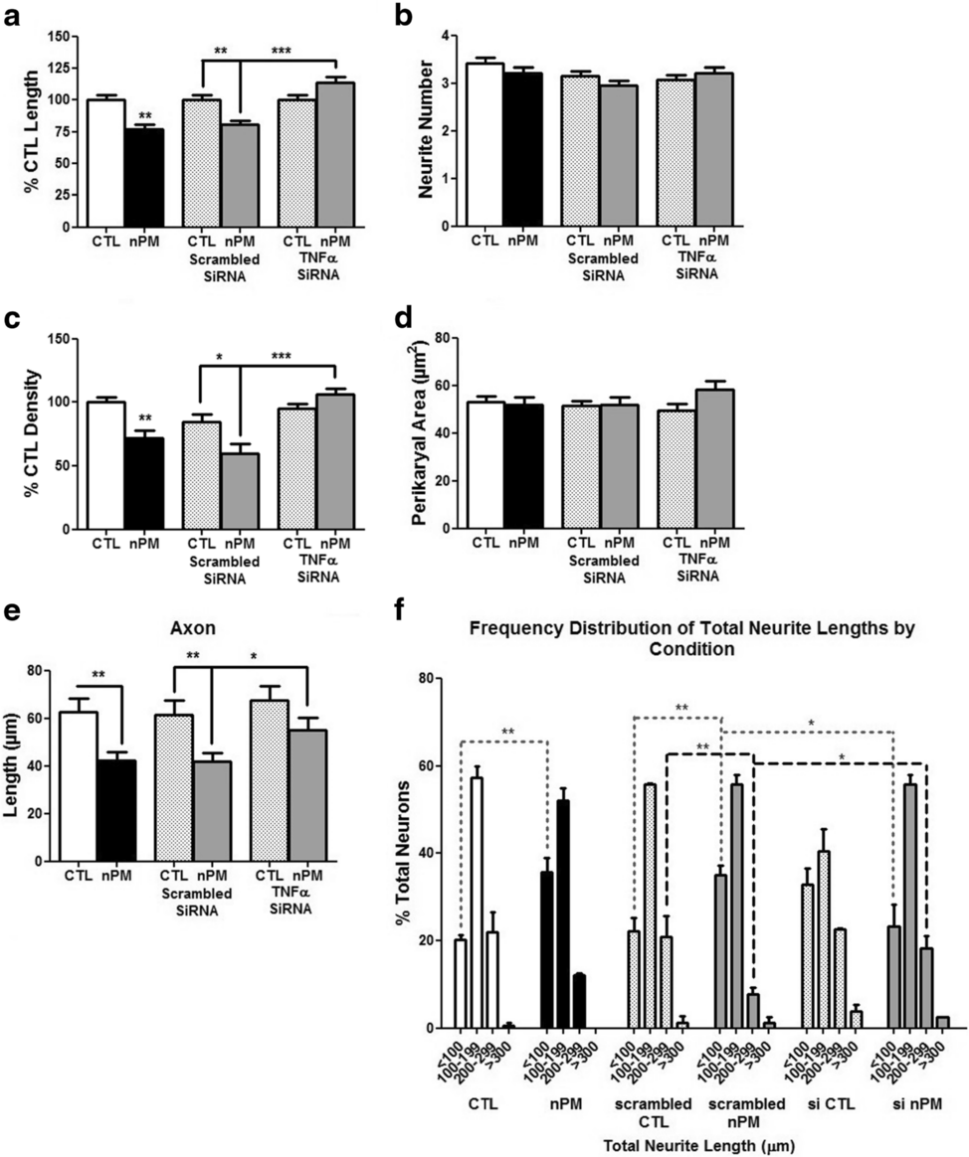
TNFα siRNA rescued the inhibition of neurite outgrowth in nPM-CM. **a** CM from glia cultures transfected with siRNA to TNFα showed a rescue of total neurite outgrowth vs control cultures transfected with scrambled siRNA and treated with nPM (**p < 0.01, ***p < 0.001; ANOVA with Tukey’s post-test). **b** Neurite number, not altered by treatment. **c** Neurite-associated βIII-tubulin changed in parallel with total neurite length. **d** Neuronal perikaryal area, not altered by treatment. **e** Mean axon length shortening by nPM treatment was rescued with TNFα siRNA. **f** Neurons treated with nPM or scrambled siRNA + nPM mixed glial CM had shorter neurites (**p < 0.01; two-way ANOVA with Tukey’s post-test; avg. three experiments)

To define the role of TNFα in nPM-CM in neurite outgrowth inhibition, mixed glia were transfected with TNFα siRNA, which reduced TNFα mRNA by 70 % vs scrambled siRNA control (not shown). CM from TNFα siRNA-treated glia (also nPM exposed) rescued neurite outgrowth, total neurite density, and axon length (Fig. 5a, c, e), but without altering total neurite number or the area of neuronal perikarya (Fig. 5b, d). The frequency distribution of total neurite lengths showed consistent shortening: 35 % of neurons grown in nPM or nPM + scrambled siRNA glial media had total neurite lengths <100 μm vs 20 % in control or TNFα-silenced conditions. Only 10 % of these neurons had total neurite lengths >200 μm vs 20 % of control and siRNA treatments (Fig. 5f).

**Fig. 6:**
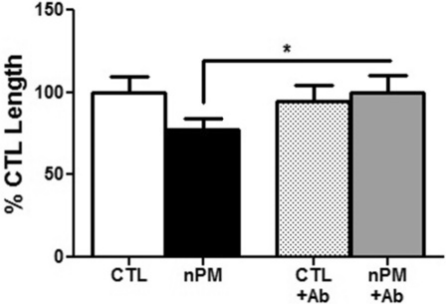
Immunoneutralization of TNFα in CM rescued nPM inhibition of neurite outgrowth. Neurite outgrowth was inhibited by 25 % in CM from nPM-exposed mixed glia (nPM-CM) (*p < 0.05; ANOVA). TNFα immunoneutralization of CM rescued neurite length

The role of TNFα in the nPM-CM was further shown by immunoneutralization with anti-TNFα antibodies, which also rescued neurite outgrowth (Fig. 6).

### Blocking TNFR1 in neurons reduced the CM effect on neurite outgrowth

**Fig. 7:**
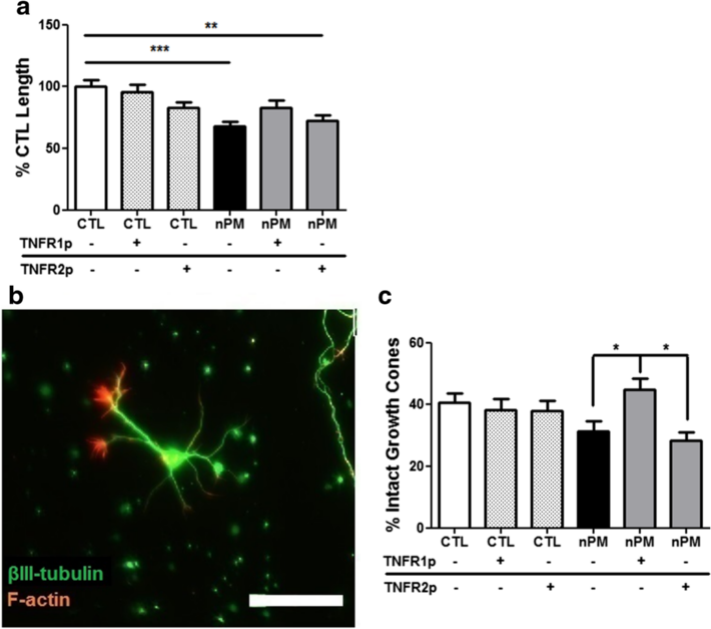
TNFR1-blocking peptide rescued neurite outgrowth and growth cone collapse. **a** Pre-incubation of neurons with TNFR1-blocking peptide (TNFR1p) before exposure to nPM-CM restored neurite outgrowth to control levels. Pre-incubation with TNFR2-blocking peptide (TNFR2p) did not alter the nPM effect on neurite outgrowth. (**p < 0.01; ***p < 0.001; ANOVA with Tukey’s post-test; *n* = 50). **b** Representative neuron with two intact and five collapsed growth cones. Intact growth cones have lamellipodia and multiple filopodia. *Scale bar* = 50 μm. **c** Blocking TNFR1 inhibited the nPM effect on percent intact growth cones per neuron (*p < 0.05; ANOVA)

The role of TNFα receptors was evaluated by “blocking peptides” (antibodies) to the C terminus of their respective TNFRs. Neurons were pre-incubated with blocking peptides before application of nPM-CM. The anti-TNFR1 peptide restored total neurite length to control levels, while anti-TNFR2 had no effect on the nPM-CM inhibition (Fig. 7a). Growth cones were increased 30 % only by anti-TNFR1 (Fig. 7b, c), with 10 % fewer neurites <100 μm vs nPM-CM-treated cultures (data not shown).

## Discussion

These studies further document the role of glial TNFα in neuroinflammatory responses to air pollution PM that modify neuronal function. In particular, we studied nPM, which are a subfraction of urban PM2.5 (“Methods” section) that epidemiological studies have associated with neurodevelopmental dysfunctions from pre- and early childhood exposure [37, 38]. Rodent models include exposure of pregnant rats to nPM, which altered neonatal neuronal maturation [39] and exposure of early postnatal mice to ultrafine PM, which caused ventriculomegaly and glial activation [22]. For inflammatory responses, we focused on TNFα because of its consistent elevation in rodent models of air pollution [8, 10, 40–42] as well as in postmortem human brains from a highly polluted megacity [15]. In vitro activities of nPM include induction of TNFα in mixed glia from cerebral cortex and reduced neurotrophic support by the CM of mixed glia exposed to nPM [10]. We also document the stability of nPM activity to induce TNFα, in which the dose response was nearly identical, despite collection from the same site on different years.

We hypothesized that glial TNFα was a mediator of these CM effects because TNFα in vitro inhibits neurite outgrowth [24, 34] with growth cone collapse [43] and inhibits astrocytic neurotrophic support [44]. Before further analysis of cerebral cortex glia, we investigated if TNFα induction by air pollution PM extended to the OE which is the initial site of exposure of inhaled air pollutants from which olfactory neurons project into the brain. Importantly, besides the acute inflammatory responses of TNFα and macrophage activation, the OE expresses high levels of phase I and phase II detoxifying enzymes, e.g., cytochrome P450 (CYP) isoforms and glutathione S-transferases (GST) [45, 46], which may mediate detoxifying environmental pollutants.

We developed an ex vivo model for the initial impact of air pollution on olfactory neurons, in which the neonatal mouse nose is incubated with aqueous suspensions of nPM. During ex vivo incubation with nPM, the neonatal OE showed rapid shrinkage of the OSN dendritic layer concurrently with induction of TNFα and macrophage activation in the OE. We hypothesized that olfactory neuron dendritic regression was driven by TNFα from macrophages in the OE. This is supported by another model of olfactory damage, where TNFα was shown to inhibit OE regeneration [47]. We further tested this hypothesis with primary glial cultures from the neonatal mouse cerebral cortex as discussed below.

In rodent models, nPM cross from the nose into the brain by undefined transport processes which are presumed to include the projections of OSN axons that synapse in the main olfactory bulb [19, 20]. Studies with different artificial ultrafine PM observed that inhaled [19] or nasally instilled [20] PM reached the forebrain and cerebellum as well as the OB within 24 h [48]. The passage of nPM from the nares beyond the OB into the posterior brain structures gives a rationale for using cerebral cortex glia as an experimental model for direct nPM exposure. Although astrocyte cell bodies were not detected in the OE, there still may be a role of astrocytic TNFα in the OB which has deep neuronal projections caudally into the brain.

To develop our observations of OE dendritic shrinkage, we further analyzed mechanisms of neuronal responses to nPM with a model of primary cultures of mixed glia and neurons from the cerebral cortex. We extended our observation that CM from nPM-exposed mixed glia inhibited neurite outgrowth [10] by resolving cell type contributions. In subcultures from mixed glia, microglia contributed 60 % of the TNFα in CM, consistent with the greater inhibition of neurite outgrowth by CM from microglia. Similarly, the microglial CM caused more inhibition of neurite outgrowth and neurite density than the astrocyte CM. A primary role of microglia in nPM responses is also consistent with the low abundance of GFAP-immunopositive cells or processes in the OE, especially during development [49]. The precise mechanism of nPM uptake in cells is not well defined but could include phagocytosis [50] as well as direct diffusion [51].

The role of TNFα in neurite outgrowth inhibition was further defined by suppressing TNFα expression with siRNA, by immunoblockade of TNFα, and by TNFR1 blockade, all of which restored neurite outgrowth to control levels. The restoration of axonal length by TNFα immunoblockade is also consistent with enhanced axonal regeneration by TNFα blockade after injury [34]. Because these conditions did not consistently alter the total number of neurites or neuronal perikaryal size, they define an experimental model for effects of nPM on neuronal plasticity without major cell damage that could be useful for efficient screening of neuroprotective agents.

Several mechanisms may mediate the glial-derived TNFα influences on neurite outgrowth. Although TNFα has both cytosolic and transmembrane forms, we would not expect a significant role for transmembrane TNFα because the nPM-CM has negligible cell membrane content. Notably, of the two defined TNFRs, only blockade of TNFR1 rescued the nPM-CM effect. This specificity is consistent with the 20-fold higher affinity of TNFR1 (K_a_) to soluble TNFα vs TNFR2 [52–54]. TNFR1 activation is associated with reduced neuronal differentiation, as well as apoptosis, whereas TNFR2 is associated with neuroprotection and survival [55]. Blocking TNFR1 may have improved neurite outgrowth by diminishing growth cone collapse (Fig. 7c) through reduction of CM TNFα signaling. The small GTPase RhoA mediates the TNFα inhibition of neurite outgrowth [24], but mechanisms from receptor signaling to neurite outgrowth inhibition are less defined. RhoA activation by TNFα can cause growth cone collapse and attenuate neurite outgrowth [24, 34, 56], but this process has not been directly linked to TNFR1/2 signaling [23].

## Conclusions

These experimental findings suggest a role for TNFα induction by the nPM subfraction of PM2.5. We propose that TNFα from microglia-macrophage activation by nPM in inhaled air pollutants is a main mediator of neuroinflammation and neurodevelopmental impairments from airborne particulate pollution. Studies are needed to evaluate other TNF superfamily receptors and their relation to the glutamatergic changes observed in rodent models of air pollution [10, 11, 42]. Further fractionation of the nPM may resolve the role of the persistent free radicals in nPM [10] and specific chemical components in the heterogeneous nPM. Although these nPM fractions do not include ozone and other gases with cognitive epidemiological associations [2, 21], gaseous pollutants could still contribute to nPM neurotoxicity in the real world. Identifying the neurotoxic components in air pollution could prioritize environmental policy targets to minimize neurodegenerative activities in the urban air we must breathe.

## Abbreviations

CM: conditioned media
CTL: control
DL: dendritic layer
GFAP: glial fibrillary acidic protein
Iba1: ionized calcium-binding adaptor molecule 1 and monocytic marker
IHC: immunohistochemistry
IL-1: interleukin 1
nPM: nano-sized particulate matter
PM: particulate matter
OB: olfactory bulb
OE: olfactory neuroepithelium
OSN: olfactory sensory neuron
TNFα: tumor necrosis factor alpha
TNFR: tumor necrosis factor receptor
